# Supplementary material for: Lumbo-pelvic proprioception in sitting is impaired in subgroups of low back pain–But the clinical utility of the differences is unclear. A systematic review and meta-analysis
Source: PLoS One. 2021 Apr 26;16(4):e0250673. doi: 10.1371/journal.pone.0250673 (PMC8075231; doi:10.1371/journal.pone.0250673)
Supplement: S2 File — (PDF) [file pone.0250673.s003.pdf]

Online supplementary material 1:

Electronic database search strategy in PubMed

(((((((((((((((((((((((Differential Threshold) OR joint sense) OR just noticeable difference) OR Kinesthesis) OR Kinesthes*) OR kinesthetic perception) OR mechanoreceptors) OR movement sense) OR motion sense) OR Muscle Spindles) OR Muscle Spindle) OR position sense) OR propriocept*) OR proprioception) OR repositioning) OR reposition*))

AND

((((((((((((((((((((((((Spondylosis[Title/Abstract]) OR Sciatic Neuropathy[Title/Abstract]) OR Sciatica[Title/Abstract]) OR lumbar trouble[Title/Abstract]) OR lumbar symptoms[Title/Abstract]) OR lumbar pain[Title/Abstract]) OR lumbar injur*[Title/Abstract]) OR lumbar dysfunction[Title/Abstract]) OR lumbar disorder*[Title/Abstract]) OR lumbar complaints[Title/Abstract]) OR (low back pain[Title/Abstract] OR low-back pain[Title/Abstract])) OR lumbago[Title/Abstract]) OR dorsalgia[Title/Abstract]) OR coccydynia[Title/Abstract]) OR Coccyx[Title/Abstract]) OR back trouble[Title/Abstract]) OR back symptoms[Title/Abstract]) OR Back Pain[Title/Abstract]) OR back injur*[Title/Abstract]) OR Back Injuries[Title/Abstract]) OR back dysfunction[Title/Abstract]) OR back disorder[Title/Abstract]) OR back complaints[Title/Abstract]) OR backache[Title/Abstract])) AND Humans[Mesh]))

NOT

(surgery[Title/Abstract] OR systematic review[Title/Abstract])) AND Humans[Mesh])) NOT (((((neck pain AND Humans[Mesh])) OR (spine surgery AND Humans[Mesh])) OR (cauda equina AND Humans[Mesh])) AND Humans[Mesh])

Filters: Humans
