# Supplementary material for: Lumbo-pelvic proprioception in sitting is impaired in subgroups of low back pain–But the clinical utility of the differences is unclear. A systematic review and meta-analysis
Source: PLoS One. 2021 Apr 26;16(4):e0250673. doi: 10.1371/journal.pone.0250673 (PMC8075231; doi:10.1371/journal.pone.0250673)
Supplement: Attachment — Submitted filename: Response to Reviewers.docx [file pone.0250673.s004.docx]

| **Editor’s and Reviewers’ comments** | **Authors’ responses** |
| --- | --- |
| Journal requirements:  When submitting your revision, we need you to address these additional requirements.  1.) Please ensure that your manuscript meets PLOS ONE's style requirements, including those for file naming. The PLOS ONE style templates can be found at  https://journals.plos.org/plosone/s/file?id=wjVg/PLOSOne_formatting_sample_main_body.pdf and  https://journals.plos.org/plosone/s/file?id=ba62/PLOSOne_formatting_sample_title_authors_affiliations.pdf | We have checked PLOS ONE’s style requirements and our submission conforms with these. |
| 2.) Thank you for including the statement that "PubMed, Cochrane, CINAHL, EMBASE and Web of Science databases were independently searched by two reviewers (VK and YS) from inception of database to 28 March 2020 without language restriction, with aim to reduce language and publication bias. ". Please revise this statement to clarify whether all databases were searched from inception, or if there were any limits placed on the publication dates in your search. | The statement was revised as suggested. |
| 3.) In the methods, please describe how risk of bias was assessed in individual studies (including specification of whether this was done at the study or outcome level, or both, and the specific test employed, such as the I^2 statistic), and how this information was used in any data synthesis. | We have added all the requested information for the risk of bias assessment in the “Methods – Risk of Bias” section of the manuscript (lines 154-175).  All the requested information for the specific tests employed and how this information was used in any data synthesis, is described in “Data analysis, synthesis, and summary of findings” and “Assessment of the quality of the body of evidence” sections of the manuscript. We have also edited the sections for clarity. |
| 4.) In your Data Availability statement, you have not specified where the minimal data set underlying the results described in your manuscript can be found. PLOS defines a study's minimal data set as the underlying data used to reach the conclusions drawn in the manuscript and any additional data required to replicate the reported study findings in their entirety. All PLOS journals require that the minimal data set be made fully available. For more information about our data policy, please see <http://journals.plos.org/plosone/s/data-availability>.  Upon re-submitting your revised manuscript, please upload your study’s minimal underlying data set as either Supporting Information files or to a stable, public repository and include the relevant URLs, DOIs, or accession numbers within your revised cover letter. For a list of acceptable repositories, please see http://journals.plos.org/plosone/s/data-availability#loc-recommended-repositories. Any potentially identifying patient information must be fully anonymized. We will update your Data Availability statement to reflect the information you provide in your cover letter. | We have now uploaded our study’s minimal underlying data set as a Supporting Information file. |
| 5.) Please note that in order to use the direct billing option the corresponding author must be affiliated with the chosen institute. Please either amend your manuscript to change the affiliation or corresponding author, or email us at plosone@plos.org with a request to remove this option. | We have recently published two papers in Plos One and used the direct billing option.  Also, after contacting (28th January 2021) the Qatar National Library (Open access author fund) their response was the following: “The Qatar National Library has an agreement with PLOS, which allows the Library to directly pay article-processing charges (APCs) for accepted open access publications.” |
| 6.) Please include captions for your Supporting Information files at the end of your manuscript, and update any in-text citations to match accordingly. Please see our Supporting Information guidelines for more information: http://journals.plos.org/plosone/s/supporting-information. | The captions for all supporting Information files have been added at the end of the manuscript. |
| **Reviewer #1** | **Responses to reviewer #1** |
| The authors have prepared a thoughtful and necessary review on lumbopelvic hip proprioception in a sitting posture in non-specific low back pain. This is a great update to review and provide a meta-analysis of the seated outcomes since the most recent Tong et al, 2017 review that was mixed on standing and seated proprioception. The inclusion of subgrouping as a secondary aim allowed for some unique analyses that shed light on clinical application. | We would like to thank the reviewer for the feedback. |
| Abstract: Well organized and informative, although clarity on use of “low back pain” as the same as “non-specific low back pain” is important to define from the outset. This would also strengthen the inclusion of the secondary objective of the review. | We have changed “low back pain” into “non-specific low back pain” in the abstract for clarity, as suggested. |
| Sitting is not mentioned in the objective or as part of the active repositioning in the search strategy portion of the methods.  As this is central to the review and meta-analyses (and in the title) the findings and quality of evidence should both be included here along with this. | We thank the reviewer for this comment. We have tried to address this throughout the revised abstract, to better reflect the centrality of the seated posture to this study. |
| Directional subgrouping pattern needs a definition (may not have space within abstract, but is assumed throughout review) | We have edited the introduction of the manuscript providing evidence that the NSLBP is not a homogenous group, a definition, and a brief explanation of the directional pattern classification system. |
| Lines 55-62: The introduction begins with the same sentences as the abstract, except (NSLBP) is included in the introduction. These sentences should not be an exact copy. | Following your recommendation, the sentence in introduction has been edited. |
| [The inclusion of this abbreviation addresses the confusion of using NSLBP and LBP interchangeably earlier in the abstract.] | We have changed “low back pain” into “non-specific low back pain” in the abstract for clarity, as suggested. |
| A brief definition of NSLBP would be helpful to include in the opening paragraph, perhaps with the sentence indicating "proprioceptive deficits might be associated with the underlying characteristics and mechanisms of NSLBP development”. The connection between the two and need for the review could be stressed further beyond the described lack of separation of seated proprioceptive assessment and intervention in prior reviews. This will strengthen and could link to the clinical implication discussion at the end of the review surrounding the MCID. | We thank the reviewer for the recommendation. We have edited the opening paragraph providing a definition of non-specific low back pain. |
| Lines 69-72: Including the level of evidence of these studies would further strengthen this statement. | We have edited the paragraph to reflect that the level of evidence was low, by referring to the study design. |
| Lines 96-97: Item (a) reads as exclusion criteria, could be reworded simply as “systematic reviews and case studies were excluded”. Were case series also excluded? | We have edited the “types of studies and participants” section of the manuscript for clarity. |
| Lind 104: Listing some of the serious pathologies from the Tong review referenced would be useful for clarity especially with a diagnosis of exclusion (NSLBP) as the focus of the review. | We have added specific spinal pathologies from the Tong et al, 2017 review, as suggested. |
| Lines 151-152: Can the authors add a reference for the SMD effect scale? | The reference is added. |
| Lines 175-183: I agree with the authors reasoning behind the MCID establishment, although including other references that assisted in arriving at this conclusion would bolster the decision-making process that is fairly important for this review. This links back to my earlier comment from the introduction and to a later comment from the discussion. Establishing the reasoning that is given (more context was given in the discussion for this decision) throughout gives stronger purpose to this review overall. | We thank the reviewer for this suggestion. We have edited (introduction and data analysis section) and added references to the paragraph to form a stronger rationale for our decision-making process. |
| Lines 189-197: The current sentence structure is difficult to follow. If it was broken into two sentences explaining criteria for downgrading in the second sentence, it may be easier to follow. | We have edited the sentence for clarity. |
| Lines 202-203: Figure 1 explains the process well, but this sentence is somewhat misleading by indicating the search went from 3,137 and after duplicates were removed, only 16 remained that met inclusion. This could be reworded to better explain what Figure 1 states about the authors did throughout the review process to ultimately get to the 16 included studies, or simply direct the reader to Figure 1. | We have edited the paragraph for clarity. |
| Lines 216-217: The BMI or body fat % difference is mentioned from 2 of the studies, but not included in Table 1 that I can see in the total sample column. The values associated with the differences, although not primary outcomes, since they are highlighted here should be included. The age difference mentioned across 2 studies could easily be noted in Table 1 as means are included already. This becomes important with adolescent analysis later on. May want to indicate adolescent values specifically due to that separation in analyses? | Based on both reviewers’ comments, we have extensively edited Table 1. The table now provides information for the age, BMI, and disability measures of the participants. We have removed the “Results” of individual studies, as suggested from another reviewer. Also, the table is now divided into sections based on the age of the participants (adults or adolescents) and the plane of the repositioning task (sagittal or transverse plane). |
| Line 223: Recent history for controls varied across the included studies just as the NSLBP timing and at least a range of time would be beneficial to match the robust level that the NSLBP inclusion was explored in the following sentence (which was well done). | We thank the reviewer for the valid comment. We have updated the section for NSLBP (two studies were not listed), as well adding the requested information for the asymptomatic control group. |
| Lines 241-244: How were the Lam and Maffey-Ward articles considered in this part of the assessment since they were not designed for a group comparison? These seem to be a clear exception to the rule in many aspects, which is addressed well in some ways (lines 271-219, Table 2) but not clear in others since they are different studies by the same group merged for analysis. | We thank the reviewer for the comment and the opportunity to clarify our decision to combine the studies. In the study of Lam et al, 1999, the authors stated that the studies were identical in term of methodology, they tried to recruit an age-matched LBP group with controls and made comparisons by using the actual data from the previous study.  We have attached below statements from Lam et al study that drove us to make such a decision.    a) “*By viewing the magnitude of the error recorded from patients with low back pain against the results of the study by Maffey-Ward et al, 1996 of asymptomatic subjects, our study helps assess the potential sensitivity and therefore usefulness of the test to detect kinesthetic deficits in patients with low back pain*” – making clear their aim to combine study results.  b) “*A young age group of subjects with back pain was selected to eliminate the effect of aging on kinesthetic sensibility and also to approximate the age of the group studied by Maffey-Ward et al, 1996*” – The age-matching with the Maffey-Ward et al, showing clearly that they aimed to replicate the study in a LBP population.  c) “*In our study, we replicated the lumbar spine repositioning test, instrumentation, and measurements as described by Maffey-Ward et al, 1996*” – By a close observation of the methodology used, it was evident that the two studies were identical.  d) “*When the results of this study are viewed with those obtained from nonimpaired subjects (Maffey-Ward et al, 1996) (Table 2), it can be observed that the repositioning errors of the 2 populations are of a similar magnitude*” – They presented data from previous study and made comparisons and inferences. |
| Lines 252-260: Separating out with "Included and excluded studies” subheadings for each outcome becomes very tedious throughout the remainder of the review. The sentence(s) that state how many studies were included in each section could simply be stated at the beginning of each section without that additional subheading to streamline the sections. It is not consistent throughout each subheading, so removing it altogether from the sections where it is present could be helpful. | We have removed the "Included and excluded studies” subheadings for each outcome following your suggestion. |
| Lines 263-264: Mild or moderate NSLBP symptoms are used to subgroup and mild was defined with Oswestry Disability Index and Roland Morris scores, (<15 ODI and <5 Roland Morris) however moderate was not clearly defined with a score range. Also, would a mild rating include 0 on either questionnaire since that is less than 15 and 5? | We thank the reviewer that gives us the opportunity with this comment to justify our subgrouping rationale. Upon inspection of the disability scores provided by the included studies, we observed a wide range of disability scores on both Oswestry Disability Index and Roland Morris Disability Questionnaire. However, none of the scores was lower than 5. With regards to ODI, cut-off subgrouping values have been reported in the literature, though to our knowledge such values have not been established in the literature for RMDQ. Given the former, we arbitrarily decided to use these cut-off values in order to subgroup studies/participants and proceed with a subgroup quantitative synthesis.  We have now added more information in the “Data analysis, synthesis, and summary of findings” section of the manuscript for clarity.  The arbitrary selection of cut-off values has been added as a limitation of our review. |
| Line 282: The authors begin excluding adolescents and it is unclear if this is another aim altogether at this point or done intentionally for organization with the subheadings. Perhaps if the age differences were highlighted in the tables as they are mentioned earlier, this exclusion from analysis to show significant effects would be clear without an extensive additional explanation. | We thank the reviewer for this comment that will add clarity to our data analyses. We have now highlighted in the tables the age differences, as well as we added this sub-group analysis in the “Data analysis, synthesis, and summary of findings” section of the manuscript (lines 200-206). |
| The discussion is touched on with the discrepancies between adolescents and adults, (lines 550-553, 559-563) yet could be emphasized based on findings within directional subgroup. | We have extensively edited this part of the discussion with aim to emphasize the differences between adult and adolescent directional subgroups. |
| Lines 378-379: Throughout the results, as there are many outcomes to describe, the structure becomes very redundant. The authors do a very nice job in many places of describing directional results in active voice, although some places only state significance or an effect, which is only part of the story. The impactful part of the results is the directional piece and understanding the overall findings for each outcome after the analyses. Removal of the unnecessary subheadings will help with the choppiness of these sections as well. | Following your recommendation and another reviewer’s comments, we extensively revised the results section of the manuscript, by removing subheadings, reducing less relevant or redundant information, and adding description of the directional results. |
| Line 583: This is a major highlight and unclear why MCID calculation could happen in discussion based on some of the data, but was not formally included earlier. Although not all studies included had reliability data, there were other outcomes reported that were incomplete. | We thank the reviewer for pointing this out. We have now included in the methods section (Data analysis, synthesis, and summary of findings) how we calculated the cut-off value of real difference or MCID for more clarity. |
| Discussion: An extended discussion based on methodological discrepancies between NSLBP and controls primary aim and the reasons that led to outliers in each analysis would enrich the section. I agree with the authors when they state methodological diversity made concrete conclusions unsafe, although further discussion of why and how that happened with the compilation of these 16 studies could happen. Although the evidence revealed very low and low certainty in the primary aim, the discussion of this matter pulling examples from more of the included studies would improve the discussion section. The participants, interventions, measurement, specific outcomes, reporting differences and even lack of data availability played into the decisions in each analysis and ultimately affected the SMDs. The focus seemed shift heavily to the subgroup secondary aim, which was great information but not the primary aim of the review. | We have extensively edited the discussion section to address your suggestions. |
| **Reviewer #2:** | **Authors’ replies** |
| INTRODUCTION:  The introduction is concrete, which is appreciated. However, it would be advisable to include a paragraph about the systematic reviews that have been done on low back pain, and the novelty of the present review compared to previous ones. | We thank the reviewer for the feedback and the suggestions.  We have added this info in the introduction. |
| On the other hand, it is advisable that when it is mentioned that a systematic review has been done, "with meta-analysis" should be included (e.g., line 73). | We have changed the sentence accordingly. |
| METHOD:  The method is very specific, which is appreciated as it allows for replicability of the study. | We thank the reviewer for the feedback. |
| However, it is advised to include in the manuscript the keywords and search formula in the databases, including Boolean operators. | We thank the reviewer for the suggestion; however, these details are presented in Supporting Information Table. Given the length of the manuscript we are happy to abide with the editorial team’s decision. |
| RESULTS:  The results section includes a lot of information, which is normal in a systematic review with meta-analysis, but needs to improve in order and summarise the information presented in order to be easily followed. | Following your recommendation and another reviewer’s comments, we extensively revised the results section of the manuscript, by removing subheadings, reducing non-relevant or redundant information, and adding description of the directional results. |
| Thus, it is advisable to make a first table (Table 1) where by thematic areas the reader can see what has been done in each study, excluding the results of this table, as in fact they are not the expected results of a meta-analysis and the results analysed by the meta-analysis are perfectly shown in the following tables. It is recommended for this table 1 to be divided into section by subject. | Based on both reviewers’ comments, we have extensively edited Table 1. The table now provides information for the age, BMI, and disability measures of the participants, as suggested from another reviewer. We have removed the “Results” of individual studies. Also, the table is now divided into sections based on the age of the participants (adults or adolescents) and the plane of the repositioning task (sagittal or transverse plane), following your recommendation. |
| DISCUSSION:  The discussion is adequate to the results and the bibliographic depth required for explanations to the findings. | We thank the reviewer for the feedback. |
| CONCLUSION:  The conclusion is adequate and responds to the objective. | We thank the reviewer for the feedback. |
